# *Capnocytophaga canimorsus* Capsular Serovar and Disease Severity, Helsinki Hospital District, Finland, 2000–2017

**DOI:** 10.3201/eid2412.172060

**Published:** 2018-12

**Authors:** Estelle Hess, Francesco Renzi, Panu Karhunen, Mélanie Dol, Adrien Lefèvre, Jenni Antikainen, Elodie Carlier, Johanna Hästbacka, Guy R. Cornelis

**Affiliations:** University of Namur, Namur, Belgium (E. Hess, F. Renzi, M. Dol, A. Lefèvre, E. Carlier, G.R. Cornelis);; University of Eastern Finland, Kuopio, Finland (P. Karhunen);; University of Helsinki and Helsinki University Hospital, Helsinki, Finland (J. Antikainen, J. Hästbacka)

**Keywords:** dog commensal, *Capnocytophaga canimorsus*, human infection, sepsis, septic shock, typing, virulence, bacteria, zoonoses, Helsinki, Helsinki Hospital District, Finland, capsular serovar, disease severity, capsular typing, 16S rDNA sequencing, serovar J, serovar K, coagulopathy, dogs

## Abstract

We assembled a collection of 73 *Capnocytophaga canimorsus* isolates obtained from blood cultures taken from patients treated at Helsinki University Hospital (Helsinki, Finland) during 2000–2017. We serotyped these isolates by PCR and Western blot and attempted to correlate pathogen serovar with patient characteristics. Our analyses showed, in agreement with previous research, that 3 *C. canimorsus* serovars (A–C) caused most (91.8%) human infections, despite constituting only 7.6% of isolates found in dogs. The 3 fatalities that occurred in our cohort were equally represented by these serovars. We found 2 untypeable isolates, which we designated serovars J and K. We did not detect an association between serovar and disease severity, immune status, alcohol abuse, or smoking status, but dog bites occurred more frequently among patients infected with non-A–C serovars. Future research is needed to confirm serovar virulence and develop strategies to reduce risk for these infections in humans.

*Capnocytophaga canimorsus* is a gram-negative, rod-shaped, usually commensal bacteria of dog and cat oral flora that causes rare but potentially severe infections in humans (*1*,*2*). Even with administration of adequate antimicrobial therapy, *C. canimorsus*–induced septicemia can progress to a debilitating disease or septic shock and can cause a mortality rate as high as 30%. Annual incidence of *C. canimorsus* infections has been estimated at 0.5–0.67 cases/1 million persons (*3*,*4*), but in a retrospective study, a prevalence of 4.1 cases/1 million persons was estimated (*5*); this discrepancy probably resulted from the choice of diagnostics. The clinical manifestation of *C. canimorsus* infection might be mild, with influenza-like symptoms and intestinal complaints (*1*), a disease severity not always reaching the threshold for a blood culture. Moreover, *C. canimorsus* is a fastidious and slow-growing organism, rendering its culture and isolation difficult (*2*).

Human exposure to a dog’s oral flora can occur through a bite or scratch or even through just being in close proximity to the animal (*1*,*5*). Although splenectomy, asplenia, alcohol abuse, smoking, and advanced age are often described as predisposing factors for severe illness caused by this bacterium, up to 40% of patients have no obvious risk factor (*1*); thus, *C. canimorsus* should not be considered exclusively an opportunistic pathogen.

*C. canimorsus* is enveloped by a lipooligosaccharide and a capsule consisting of units of the same O antigen but assembled by different polymerases (*6*). The capsule confers to *C. canimorsus* resistance to the bactericidal effects of human serum and phagocytosis by macrophages (*6*). One study showed that despite the seemingly vast repertoire of capsular serovars among *C. canimorsus* isolates from dog mouths, 3 serovars (A, B, and C) are associated with most human infections (*7*). However, this finding was from a study carried out with just 25 isolates from patients worldwide. To validate this finding, we evaluated the serovars present in a collection of 73 isolates from patients treated at Helsinki University Hospital (Helsinki, Finland) during 2000–2017.

## Materials and Methods

### Study Setting

HUSLAB (Helsinki) is a central laboratory that offers microbiological services to the whole Helsinki Hospital District, which encompasses the city of Helsinki and surrounding municipalities. The laboratory maintains a frozen archive of bacterial isolates obtained from patient blood cultures. For the purposes of this study, we searched laboratory records for blood cultures positive for *C. canimorsus* during 2000–2017; a corresponding frozen bacteria isolate could be found for 78 patients. Of these frozen isolates, we could grow and analyze 73. To correlate analyses with clinical data, we searched patient journals, electronic patient records, and laboratory databases for patient characteristics, clinical information, and laboratory data. We recorded patient age, sex, concurrent medical conditions, medications administered, immune status, lifestyle factors, and type of contact with dogs (bitten, contact but not bitten, or not known), whenever the information was available. Of the clinical data, we recorded the level of care, length of stay in the hospital, complications, 30-day and 1-year mortality rates, and registered coagulation and fibrinolysis laboratory variables. We analyzed partial thromboplastin time according to the Owren method (*8*).

The Administrative Department of Helsinki Hospital District and Helsinki City College of Social and Health Care gave appraisal for obtaining this data from patient medical records. Because only data registers were used for acquiring data, obtaining informed consent from patients was waived.

### Bacterial Isolates and Growth Conditions

We cultured *C. canimorsus* bacterial isolates (Table 1) obtained from HUSLAB, which were originally obtained from blood samples of patients in Finland, as described previously (*9*). In brief, we incubated aerobic and anaerobic blood culture bottles with BacT/ALERT 3D (bioMérieux, Marcy l’Etoile, France) for 6 days or until the cultures became positive. We used Gram staining and cultivated all positive samples on chocolate agar, fastidious anaerobe agar, or heart infusion agar plates. For serotyping, we grew bacteria on heart infusion agar plates (BD Difco, Franklin Lakes, NJ, USA) supplemented with 5% sheep blood (Oxoid, Basingstoke, UK) and 20 µg/mL gentamicin (Sigma-Aldrich, Darmstadt, Germany) for 48 h at 37°C with 5% CO_2_.

**Table 1:** Capsular typing of 73 *Capnocytophaga canimorsus* isolates from patient blood samples, Helsinki Hospital District, Finland, 2000–2017*

Isolates	PCR typing†							Western blot typing‡									Serovar
ABC	A	B	C	D	E	A	B	C	D	E	F	G	H	I
H11, H16, H23, H37, H39, H42, H48, H52, H56, H60, H62, H70, H74, H75, H76, H78, H80	+	+	+	–	–	–		+	–	–	ND	ND	ND	ND	ND	ND	A
H3, H4, H5, H6, H9, H14, H22, H25, H26, H30, H35, H38, H49, H50, H53, H55, H57, H58, H63, H65, H67, H68, H69, H71, H72, H73, H79	+	–	+	–	–	–		–	+	–	ND	ND	ND	ND	ND	ND	B
H27	+	–	+	–	–	–		+	+	–	ND	ND	ND	ND	ND	ND	B
H1, H7, H8, H10, H13, H15, H17, H18, H19, H20, H28, H29, H33, H34, H36, H43, H44, H45, H46, H47, H51, H59	+	–	–	+	–	–		–	–	+	ND	ND	ND	ND	ND	ND	C
H41, H64	–	–	–	–	+	–		–	–	–	+	ND	ND	ND	ND	ND	D
H31	–	–	–	–	–	+		–	–	–	ND	+	ND	ND	ND	ND	E
H21	–	–	–	–	–	–		–	–	–	–	–	–	–	–	+	I
H12, H24	–	–	–	–	–	–		–	–	–	–	–	–	–	–	–	NT

*ND, not done; NT, nontypeable. †We performed PCR capsular typing using the oligonucleotides given in Technical Appendix Table). Results were interpreted as done previously (*7*): isolates positive for PCR ABC, A, and B were typed as A, isolates positive for PCR ABC and B were typed as B, and isolates positive for PCR ABC and C were typed as C. ‡Western blot analyses on polysaccharidic structures were performed by using specific polyclonal rabbit antisera.

### *C. canimorsus* Identification by 16S rDNA Sequencing

We extracted genomic DNA directly from blood culture bottles or by boiling of a single colony (Technical Appendix Table 1). We used 4 different amplification methods involving 8 different primers to sequence 16S rDNA from bacterial isolates (Technical Appendix Tables 2, 3). When >1 primer was used to sequence a PCR product, we obtained the consensus sequence using Bioedit (https://bioedit.software.informer.com), and we analyzed sequences using BLAST (https://blast.ncbi.nlm.nih.gov/Blast.cgi).

**Table 2:** Patient demographics, clinical characteristics, and contact with dogs, Helsinki Hospital District, Finland, 2000–2017*

Characteristic	No. patients†	Value
Age, y, median (IQR)	73	55 (48.3–64.8)
Sex	73	
M		38 (52.1)
F		35 (47.9)
Immune compromised	73	7 (9.6)
Smoking	48	30 (62.5)
Alcohol abuse	49	18 (36.7)
Contact with dog	73	
Not known		21 (28.8)
Contact but not bitten		15 (20.5)
Bitten		37 (50.7)
Disease severity	70	
Regular ward or emergency department		45 (64.3)
High surveillance unit		11 (15.7)
Intensive care unit		14 (20.0)
Length of hospital stay, d, median (IQR)	62	6 (3–13.3)
Deaths at day 30	73	3 (4.1)
Deaths at 1 y	61	4 (6.6)
Amputation	73	6 (8.2)

*Values are no. (%) patients except as indicated. IQR, interquartile range. †Because of missing data, number of patients in each category varied.

**Table 3:** Coagulation and fibrinolysis laboratory variables, by *Capnocytophaga* *canimorsus* serovar and disease severity, Helsinki, Finland, 2000–2017*

Variable	Reference range	Serovar			p value	Severity of illness		p value
		A, n = 17	B, n = 28	C, n = 22		Severe, n = 25	Mild, n = 45	
Platelets, 10^9^/L	150–360	109 (29–137) [2]	109 (28–140) [1]	93 (23–166) [1]	0.98	23 (9.5–89) [0]	117 (95–154.3) [4]	<0.001
PTT, %†	70–130	56 (24–71) [10]	78 (56–86) [11]	58 (44.5–75) [12]	0.284	54 (39–66) [2]	87 (70.5–109.5) [31]	<0.001
FiDD, mg/L	<0.5	47.9 (5.05–83.8) [12]	9.1 (3.4–85.7) [19]	14.5 (4.1–80.7) [15]	0.888	32.9 (5.6–81) [5]	1.6 (0.6–74.7) [40]	0.057

*Values are given as median (interquartile range) [no. missing values] except as indicated. The comparison between patients with mild and severe courses of disease was defined by the level of care they needed. Patients with mild disease were those who were treated in a regular ward or the emergency department, and patients with severe disease were those treated in high surveillance or intensive care units. FiDD, fibrin D-dimers; PTT, partial thromboplastin time. †PTT was analyzed according to the Owren method (*8*). PTT was calculated as the ratio of the result (in seconds) from normal plasma to the result (in seconds) from the patient sample x 100.

### Antisera Production and Adsorption

The production of antisera to serovars A–I has previously been described (*7*). We produced rabbit polyclonal anti-J (against isolate H12) and anti-K (against isolate H24) likewise (*7*). Immunizations were carried out at the Centre d’Economie Rurale (Aye, Belgium). The Centre d’Economie Rurale animal welfare committee approved our animal handling protocols and procedures. We adsorbed anti-J and anti-K sera with a mixture of 25 isolates from patients (Cc1–Cc25;Technical Appendix Table 4) to obtain polyclonal antibodies specifically recognizing J or K capsular serovars. We performed adsorptions by incubating 250 µL of antiserum with 6 × 10^9^ paraformaldehyde-fixed bacteria on a rotating wheel at room temperature for >2 hours. We removed bacteria by successive centrifugations. We repeated the incubations and centrifugations 4 times. We performed capsular typing of *C. canimorsus* by Western blot, ELISA, and PCR as previously described (*7*).

### Statistical Analysis

We expressed categorical data as counts and percentages and continuous data as medians and interquartile ranges. We compared categorical data between groups by Fisher exact test. We assumed continuous data were nonnormally distributed and analyzed data using Mann-Whitney U-test for 2 groups and Kruskal-Wallis nonparametric test for >3 groups. Because of the retrospective nature of the study, many data points were unavailable for many cases (data were more complete for severely ill patients and less complete for mildly ill patients), so we provided the number of patients included in each analysis. We considered p values <0.05 statistically significant and performed analyses using SPSS version 22 (https://www.ibm.com/analytics/spss-statistics-software).

## Results

### Capsular Typing Collection of 73 Isolates from Finland

We identified the 73 isolates originating from Helsinki University Hospital (Table 1) as *C. canimorsus* through 16S rDNA sequencing (Technical Appendix Tables 1, 3). We subjected isolates to a PCR designed to detect capsular serovars A, B, and C (*7*); 67 of 73 isolates were ABC positive (Table 1; Technical Appendix Figure 1). We also typed these 67 strains using A-, B-, and C-specific PCR tests (*7*). To validate the PCR typing results, we performed Western blot analyses with polysaccharide samples of the 73 isolates using antiserum specifically recognizing A, B, or C capsular serovars (Technical Appendix Figure 2). This analysis confirmed the PCR typing results and interpretation of all isolates tested, except H27. According to Western blot analyses, isolate H27 could be considered serovar A or B, but in agreement with the PCR results, we considered this isolate a B capsular serovar only. In short, 91.8% (67/73) of isolates tested were serovars A (n = 17), B (n = 28), or C (n = 22).

**Figure 1:**
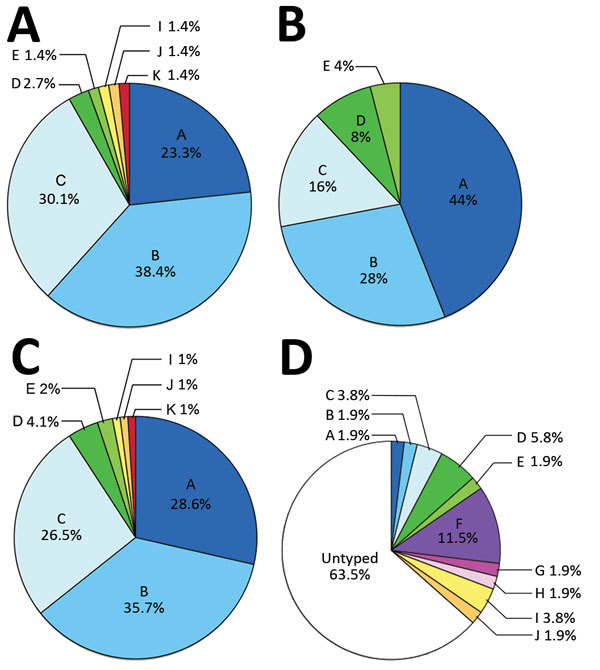
Prevalence of capsular serovars among *Capnocytophaga canimorsus* isolates from patients and dogs. A) Prevalence among 73 isolates from patients in Helsinki, Finland, 2000–2017. B) Prevalence among 25 isolates acquired from patients worldwide. C) Prevalence among pooled samples (n = 98). D) Prevalence among 52 isolates from dog mouths, Switzerland and Belgium. Percentages do not add up to 100% because of rounding. A portion of the data presented in panels B and D were previously published (*7*).

**Figure 2:**
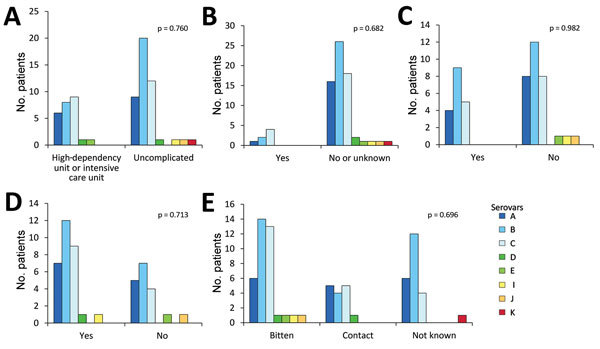
Association between *Capnocytophaga canimorsus* capsular serovar and various patient factors, Helsinki, Finland, 2000–2017. A) Disease severity (n = 70); B) immune compromised (n = 73); C) alcohol abuse (n = 49); D) smoking status (n = 48); and E) contact with dogs (n = 73). Fisher exact test was used for statistical analysis.

We then subjected isolates to PCR analyses for the detection of capsular types D and E, which have previously been detected among *C. canimorsus* isolates from human infections (*7*). Two isolates were serovar D and 1 serovar E (Technical Appendix Figure 1), findings that were confirmed by Western blot analyses (Table 1;Technical Appendix Figure 2). We tested the 3 remaining nontypeable (non-A–E) isolates by Western blot for capsular types F–I, which have only been detected in isolates obtained from dogs (*7*). Isolate H21 was typed as serovar I, leaving only 2 strains (H12 and H24) not typed of the 73 tested.

We raised rabbit antisera against H12 and H24 bacteria and adsorbed antisera with related bacteria strains. The 2 new antisera recognized only the capsule of the isolate against which they were raised, indicating the 2 isolates belonged to 2 new serovars, which we named J and K (Technical Appendix Figure 2). Thus, the 73 *C. canimorsus* isolates from the Helsinki University Hospital collection comprised 8 serovars (Figure 1, panel A); A, B, and C dominated (91.8%), consistent with the findings of the previous study involving 25 worldwide isolates (Figure 1, panel B) (*7*). The distribution of serovars A (p = 0.071), B (p = 0.47), C (p = 0.20), D (p = 0.27), E (p = 0.45), and I–K (p = 1) was not significantly different between the 2 collections (all p values analyzed by Fisher exact test; Figure 1, panel C).

### Screening of Dog Isolates for Capsular Serovars J and K

We next tested for the prevalence of the J and K capsular serovars in a previously described collection of *C. canimorsus* isolates obtained from mouths of healthy dogs (*7*,*10*). We screened these 52 dog isolates by ELISA using the antisera we produced. Although no isolates reacted with the anti-K serum, isolate CcD35 from a dog in Switzerland reacted with the anti-J serum (Figure 1, panel D;Technical Appendix Table 5). We confirmed this result by Western blot analysis of the polysaccharidic structures (Technical Appendix Figure 2), which showed that capsular serovar J is thus not limited to Finland.

### Correlation between Disease Severity and Capsular Type

We also tested the association between serovar and disease severity. For this investigation, the level of care was used as a surrogate; patients treated in a regular ward or who had only visited the emergency department were regarded as having a mild course of disease, and patients treated in a high-dependency or intensive care unit were regarded as severely ill. No statistically significant difference could be found in the proportions of any serovar between patients with mild and severe disease (p = 0.76; Figure 2, panel A). Among the 73 cases of *C. canimorsus* infection included in this study, 3 were fatal (Table 2). The isolates from these 3 patients were serovars A (H80), B (H26), and C (H28). Extensive amputations were reported in 6 cases, among which included the nonsurviving patient infected with the capsular B isolate H26. The 5 other capsular types associated with amputations were A (n = 2, H48 and H56), B (n = 1, H79), and C (n = 2, H46 and H59). Therefore, capsular serovars A, B, and C are all capable of causing severe disease in humans.

We looked for an association between capsular serovar and patient immune status or lifestyle factors but found no statistically significant link between serovar and immune compromised state (p = 0.682), alcohol abuse (p = 0.982), or smoking (p = 0.713) (Figure 2, panels B–D). We defined patients as immune compromised if they had been on immune suppressive medication or had recently received chemotherapy, had a concurrent medical condition associated with impaired immunity or active cancer, or had undergone splenectomy. One of the 2 splenectomized patients had a severe course of disease, but both survived.

Severe *C. canimorsus* infections are often associated with purpura or petechiae, disseminated intravascular coagulation, and gangrene of extremities (*1*). In particular, coagulation disorders were found to be associated with 94% of patients having *C. canimorsus*–induced septic shock in a 10-year retrospective study in Helsinki (*5*). In our study, no statistically significant association could be found between coagulation and fibrinolysis laboratory variables (platelet count, partial thromboplastin time, fibrin D-dimers) and capsular serovars (Table 3). Given the low number of cases associated with some serovars, we could assess only the 3 dominant serovars (A, B, and C). We compared coagulation and fibrinolysis disorder markers between patients with mild and severe clinical course. As expected, the analyzed variables were more affected in patients with a severe course of infection (Table 3). Of note, deviating coagulation and fibrinolysis variables were frequently present in patients with mild courses of disease, further strengthening the previously reported close association of *C. canimorsus* infection and coagulation disorders.

### Correlation between Type of Contact with Dog and Capsular Type

The type of contact with dogs did not differ among infections with any of the dominant serovars, but 4 of the 5 patients infected with serovar D, E, I, or J had been bitten (Figure 2, panel E). The contact type was not known for the patient infected with the serovar K isolate, the fifth rare serotype.

## Discussion

In this study, we analyzed 73 *C. canimorsus* isolates obtained from patients treated at Helsinki University Hospital. All isolates were serotyped and found to be endowed with a capsular polysaccharide (CPS), further confirming the commonality of the presence of a CPS in *C. canimorsus* isolates (*6*,*7*). We confirmed the high prevalence of capsular serovars A, B, and C among isolates from human infections; 67 (91.8%) of 73 isolates were typed as 1 of these 3 serovars. No significant difference was found in the prevalence of these serovars between this collection of 73 isolates from Finland and a previously studied collection of 25 isolates obtained from cases worldwide (*7*). Among the 98 *C. canimorsus* isolates from these 2 studies, 89 (90.8%) were capsular types A, B, or C. Our data confirmed that serovars A, B, and C are significantly more common among clinical isolates than dog isolates (4/52; 7.6%), suggesting these serovars are more virulent than the others. Our data also confirmed that serovars A, B, and C are present in different geographic areas.

Besides the A, B, and C serovars, the Helsinki collection contained 2 other serovars: 2 isolates of serovar D and 1 of serovar E. This observation is of high interest because serovars D and E were previously isolated from patients in the United States (n = 1), Belgium (n = 1), and Switzerland (n = 1) (*7*). Thus, although serovars D and E represent only 4.1% and 2%, respectively, of the total clinical isolates in this study, these serovars should be considered virulent and taken into account in prophylaxis.

One patient in our cohort was infected with a serovar I strain. This serovar had not been encountered before among humans but was found in dogs (1 in Belgium and 1 in Switzerland) (*7*). These findings suggest that not only serovars A–E but also rare serovars are widely distributed.

Last, we describe 2 new capsular serovars, J and K, each with a limited (1%) prevalence in human infections. We tested these 2 new antisera against our collection of isolates obtained from dogs in Switzerland and Belgium (*10*) and found 1 *C. canimorsus* isolate had a J-type CPS. Thus, using the 11 antisera we have that are specific to serovars A–K, which identified 98 human clinical isolates, we can only type 36.5% (19/52) of our collection of dog isolates from Switzerland and Belgium. This finding, again, reinforces the hypothesis of the existence of a large repertoire of CPS serovars in *C. canimorsus* among dog isolates.

Because *C. canimorsus* extensively deglycosylates human N-linked glycoproteins from cell surfaces (*11*–*13*), a given blood group might be a predisposing factor for *C. canimorsus* infection, but further research is needed to investigate an association between blood type and serovar. Blood group information was available for 55 patients in our cohort, and we found no enrichment in any blood groups among patients infected with *C. canimorsus* compared with the blood group distribution of the population of (data not shown).

The availability of clinical records associated with the isolates typed in this study gave us the opportunity to investigate the link between capsular serovar and disease severity, patient immune status, lifestyle, or type of contact with dogs. When comparing the most prevalent capsular types (A, B, and C) found in these 73 clinical isolates, we found no significant correlation between disease severity and capsular type. In the previous *C. canimorsus* capsular typing study, the authors suggested that strains belonging to capsular types of lower prevalence, like D and E, might preferentially infect immunocompromised patients (*7*); we could not draw such a conclusion here. In addition, alcohol abuse or smoking status could not be linked to infection by a specific capsular type. Alcohol abuse, smoking status, and immune suppression all were not significantly associated with disease severity or the 30-day mortality rate (data not shown), although the relatively low sample size and missing data preclude us from drawing conclusions regarding this matter.

The capsular serovars less frequently isolated in human infections, such as E, I, and J, were mainly found in patients who had been bitten, which could suggest that these serovars are less virulent than serovars A–D, perhaps requiring a deeper inoculation to provoke an infection. Unfortunately, the information on dog exposure was missing for the patient infected with the serovar K strain.

Two patients included in this study were reportedly bitten on the same day by the same dog. The isolates from these 2 patients (H44 and H46) were both typed as capsular serovar C, suggesting that the same strain infected both patients. The 2 patients had a severe form of the infection, requiring treatment in an intensive care unit. This observation of 2 patients being infected by the same dog has not been reported previously and gives an indication of the epidemiology of disease.

The observation of so few cases of *C. canimorsus* infection is indeed striking, considering that up to 74% of dogs carry *C. canimorsus* bacteria (*14*). We hypothesize that only a few *C. canimorsus* strains are virulent in humans, and few dogs carry these dangerous strains. Indeed, the 3 most prevalent serovars in human infection (A–C), represent only 7.6% of the *C. canimorsus* isolates from dogs (*7*), suggesting that a minority of dogs represent a risk for humans. This disease might be preventable in humans by identifying the dogs that carry these dangerous serotypes and specifically vaccinating them to eliminate the pathogen or drastically reduce pathogen shedding.

Technical Appendix Additional information on *Capnocytophaga canimorsus* isolates from patients and dog mouths and protocols used for analysis, Helsinki, Finland, 2000–2017.
