# Supplementary material for: Capnocytophaga canimorsus Capsular Serovar and Disease Severity, Helsinki Hospital District, Finland, 2000–2017
Source: Emerg Infect Dis. 2018 Dec;24(12):2195–201. doi: 10.3201/eid2412.172060 (PMC6256374; doi:10.3201/eid2412.172060)
Supplement: Technical Appendix — Additional information on Capnocytophaga canimorsus isolates from patients and dog mouths and protocols used for analysis, Helsinki, Finland, 2000–2017. [file 17-2060-Techapp-s1.pdf]

Article DOI: https://doi.org/10.3201/eid2412.172060

Capnocytophaga canimorsus Capsular Serovar and Disease Severity, Helsinki Hospital District, Finland, 2000–2017

Technical Appendix

Technical Appendix Table 1. Description of method and sample used for 16S rDNA identification of 73 *Capnocytophaga canimorsus* isolates from study, Helsinki, Finland, 2000–2017

Isolate ID	Method used	Sample used
H1	UNamur	Isolated colony
H3	UNamur	Isolated colony
H4	UNamur	Isolated colony
H5	HUSLAB method 3	Directly from blood culture bottle
H6	HUSLAB method 3	Directly from blood culture bottle
H7	HUSLAB method 3	Directly from blood culture bottle
H8	HUSLAB method 3	Directly from blood culture bottle
H9	HUSLAB method 3	Isolated colony
H10	HUSLAB method 3	Isolated colony
H11	HUSLAB method 3	Directly from blood culture bottle
H12	UNamur	Isolated colony
H13	HUSLAB method 3	Directly from blood culture bottle
H14	HUSLAB method 3	Directly from blood culture bottle
H15	HUSLAB method 3	Directly from blood culture bottle
H16	HUSLAB method 3	Directly from blood culture bottle
H17	HUSLAB method 3	Directly from blood culture bottle
H18	HUSLAB method 3	Directly from blood culture bottle
H19	HUSLAB method 3	Isolated colony
H20	HUSLAB method 3	Directly from blood culture bottle
H21	UNamur	Isolated colony
H22	HUSLAB method 3	Isolated colony
H23	HUSLAB method 3	Isolated colony
H24	UNamur	Isolated colony
H25	HUSLAB method 3	Directly from blood culture bottle
H26	HUSLAB method 3	Isolated colony
H27	HUSLAB method 3	Isolated colony
H28	HUSLAB method 3	Isolated colony
H29	HUSLAB method 3	Isolated colony
H30	HUSLAB method 3	Isolated colony
H31	HUSLAB method 3	Directly from blood culture bottle
H33	HUSLAB method 3	Isolated colony
H34	HUSLAB method 3	Isolated colony
H35	HUSLAB method 3	Directly from blood culture bottle
H36	HUSLAB method 3	Directly from blood culture bottle
H37	HUSLAB method 3	Directly from blood culture bottle
H38	HUSLAB method 3	Directly from blood culture bottle
H39	HUSLAB method 3	Isolated colony
H41	HUSLAB method 3	Directly from blood culture bottle
H42	HUSLAB method 3	Directly from blood culture bottle
H43	HUSLAB method 3	Directly from blood culture bottle
H44	HUSLAB method 2	Isolated colony
H45	HUSLAB method 2	Isolated colony
H46	HUSLAB method 2	Isolated colony
H47	HUSLAB method 2	Directly from blood culture bottle
H48	HUSLAB method 2	Directly from blood culture bottle
H49	HUSLAB method 1	Isolated colony
H50	HUSLAB method 1	Isolated colony
H51	HUSLAB method 1	Directly from blood culture bottle
H52	HUSLAB method 1	Directly from blood culture bottle
H53	HUSLAB method 1	Directly from blood culture bottle

Isolate ID	Method used	Sample used
H55	HUSLAB method 1	Isolated colony
H56	HUSLAB method 1	Directly from blood culture bottle
H57	HUSLAB method 1	Directly from blood culture bottle
H58	HUSLAB method 1	Directly from blood culture bottle
H59	HUSLAB method 1	Directly from blood culture bottle
H60	HUSLAB method 1	Isolated colony
H62	HUSLAB method 1	Directly from blood culture bottle
H63	HUSLAB method 1	Isolated colony
H64	HUSLAB method 1	Isolated colony
H65	HUSLAB method 1	Directly from blood culture bottle
H67	HUSLAB method 1	Directly from blood culture bottle
H68	HUSLAB method 1	Isolated colony
H69	HUSLAB method 1	Directly from blood culture bottle
H70	UNamur	Isolated colony
H71	UNamur	Isolated colony
H72	UNamur	Isolated colony
H73	UNamur	Isolated colony
H74	HUSLAB method 1	Directly from blood culture bottle
H75	HUSLAB method 1	Isolated colony
H76	UNamur	Isolated colony
H78	HUSLAB method 1	Directly from blood culture bottle
H79	HUSLAB method 1	Isolated colony
H80	HUSLAB method 1	Isolated colony

Technical Appendix Table 2. Oligonucleotides used for typing *Capnocytophaga canimorsus* isolates, Helsinki, Finland, 2000–2017

Name	Sequence 5′-3′	Reference
533R	TTACCGCGGCTGCTGGCAC	(*11*)
FD1 mod	AGAGTTTGATCYTGGYTYAG	(*11*)
CLSI-F	TTGGAGAGTTTGATCMTGGCTC	(*12*)
Forward Bosshard	AGAGTTTGATCMTGGCTCAG	(*12*)
Reverse Bosshard	GTATTACCGCGGCTGCTG	(*12*)
27F	AGAGTTTGATCCTGGCTCAG	(*13*,*14*)
1100R	GGGTTGCGCTCGTTG	(*13*,*14*)
685R	TCTACGCATTTCACCGCTAC	(*13*,*14*)
SeroA-fw	CATACCATGGGAAAAAAAGTACCAATAGTTTTTATATTTAACC	(*10*)
SeroA-rev	CCGCTCGAGTCATTTTTTTATCTTTTTTAATATATTCCAC	(*10*)
SeroB-fw	CATACCATGGGAATTAACAAAATTCTAATAG	(*10*)
SeroB-rev	CCGCTCGAGTTATTTTTTATTTTCATTAG	(*10*)
SeroC-fw	GGCGTATATCGTTGCTATTTTGTATG	(*10*)
SeroC-rev	CTATTAATATTTTCATTGTACACCACTTC	(*10*)
SeroD-fw	GATTTAAAAAATATAGTATTTTAGGAATTATCG	(*10*)
SeroD-rev	CTATACTTGTTCCCACTTTTTAGTTTC	(*10*)
SeroE-fw	GGAGGAGGAAAAGTATTATTAGATTATC	(*10*)
SeroE-rev	CTATTCATAATTCTTAAAGATACTTATCAATTC	(*10*)
SeroABC-fw	CTTGGTTAGGTAAAGTTGCCTTAC	(*10*)
SeroABC-rev	CAACATTTCTCCCATCTTATAATCCC	(*10*)

Technical Appendix Table 3. Description of 16S rDNA sequencing methods used for *Capnocytophaga canimorsus* isolates, Helsinki, Finland, 2000–2017

	Method name			
Category	HUSLAB method 1	HUSLAB method 2	HUSLAB method 3	UNamur
Forward primer	533R	CLSI F	CLSI F	27F
Reverse primer	FD1mod	Reverse Bosshard	Reverse Bosshard	1100R
DNA polymerase	AmpliTaq Gold (Applied Biosystems, Waltham, MA, USA)	AmpliTaq Gold (Applied Biosystems. Waltham, MA, USA)	MolTaq 16S (Molzym, Bremen, Germany)	Takara PrimeSTAR (Clontech, Kasatsu, Japan)
Amplification program	94°C for 10 min. 35 cycles of 94°C for 15 s, 55°C for 15 s, and 72°C for 30 s	95°C for 10 min. 30 cycles of 94°C for 15 s, 64°C for 15 s, and 72°C for 30 s	95°C for 2 min. 40 cycles of 94°C for 15 s, 64°C for 15 s, and 72°C for 30 s	5 cycles of 94°C for 30 s, 60°C for 2 min, and 72°C for 3 min with a reduction of annealing temperature of 1.5°C/cycle. 30 cycles of 94°C for 30s, 52°C for 90 s, and 72°C for 3 min. Final elongation at 72°C for 10 min.
Sequencing primer(s)	533R	Forward Bosshard	Forward Bosshard	27F, 685R, and 1100R

Technical Appendix Table 4. *Capnocytophaga canimorsus* strains used in study, Helsinki, Finland, 2000–2017*

Isolate ID	Collection	History and geographic origin	Reference
Cc1	BCCM/LMG 11511; CCUG 17234; strain P810; strain SSI P810	BCCM/LMG <CCUG Sweden <W.Frederiksen <J.Ursing. Malmö. Sweden	(*1*)
Cc2	CCUG 70775	G. Wauters and M. Delmee. Cliniques Universitaires St Luc. Brussels. Belgium	(*2*)
Cc3	–	G. Wauters and M. Delmee <Sint-Jan Hospital. Brugges. Belgium	(*3*)
Cc4	CCUG 70776	J. Schrenzel. Hopitaux Universitaires de Genève. Switzerland	(*4*)
Cc5	BCCM/LMG 28512. CCUG 70777	G. Wauters and M. Delmee <Clinic of Libramont. Libramont. Belgium	(*5*)
Cc6	CCUG 70778	KU Leuven. Leuven. Belgium	(*6*)
Cc7	–	G. Wauters and M. Delmee. <KU Leuven. Leuven. Belgium	(*5*)
Cc8	–	M. Delmee <Liège. Belgium	(*6*)
Cc9	BCCM/LMG 11510. CCUG 12569. CDC A3626	BCCM/LMG. CCUG <R. Weaver. CDC. Atlanta. Georgia <Virginia. USA	(*7*)
Cc10	BCCM/LMG 11541. CCUG 24741. ATCC 35978. CDC C8936	BCCM/LMG. MCCM. ATCC <R. Weaver. CDC. Atlanta. Georgia <California Health Department. California. USA	(*7*)
Cc11	BCCM/LMG 11551. CCUG 70779. MCCM 01373	BCCM/LMG <MCCM <A. von Graevenitz. Unersität Zurich. Switzerland	(*7*)
Cc12	ATCC 35979. CDC 7120. CCUG 53895	ATCC <R.Weaver. CDC Atlanta Georgia <California Health Dept. <San Antonio Community Hospital. California. USA	(*8*)
Cc13	–	F.S. Stals. Laurentius Ziekenhuis. Roermond. The Netherlands	(*9*)
Cc14	–	R. Jarsumbeck. Medizinisches labor Ostsachsen. Dresden. Germany	(*6*)
Cc15	–	K. Mühlemann. University Hospital Bern. Switzerland	(*6*)
Cc16	–	G. Glupczynski. Centre Hospitalier Universitaire Mont Godinne <D. Olivier. Hopital Univ. Erasme. Brussels. Belgium	(*6*)
Cc17	–	G. Glupczynski. Centre Hospitalier Universitaire Mont Godinne <D. Olivier. Hopital Univ. Erasme. Brussels. Belgium	(*6*)
Cc18	–	G. Glupczynski. Centre Hospitalier Universitaire Mont Godinne <D. Olivier. Hopital Univ. Erasme. Brussels. Belgium	(*6*)
Cc19	–	A. Magnette. Centre Hospitalier Universitaire Mont-Godinne <M Delmée <Clinique Saint Pierre. Ottignies. Belgium	(*6*)
Cc20	CCUG 55909	CCUG <E. Ek. Blood Department. PHLS. Göteborg. Sweden <UK National External Quality assessment. Colindale. London. UK	(*10*)
Cc21	CCUG 60839	CCUG <E. Ek. Blood Department. PHLS. Göteborg. Sweden	(*10*)
Cc22	CCUG 20318	CCUG <W. Frederiksen. Statens Seruminstitut. Copenhagen. Denmark	(*10*)
Cc23	CCUG 48899	CCUG <V. Roux and D. Raoult. Marseille. France	(*10*)
Cc24	CCUG 67384	CCUG <PHLS. Uddevalla <Trollhätten. Sweden	(*10*)
Cc25	CCUG 66222	CCUG <I. Adlerberth. Blood Department. PHLS. Sahlgrenska University Hospital. Göteborg. Sweden	(*10*)
CcD3-CcD106†	–	Switzerland	(*6*)
CcD113-CcD131†	–	Belgium	(*6*)

*All human isolates listed were from human septicemia cases except Cc4, which was isolated from a prosthetic aortitis case. ATCC, American Type Culture Collection; BCCM/LMG, Belgian Co-ordinated Collections of Micro-organisms, Laboratory of Microbiology, UGent; CCUG, Culture Collection University of Gothenburg; CDC, Centers for Disease Control and Prevention; MCCM, Medical Culture Collection Marburg; PHLS, Public Health Laboratory Services.
†Dog mouth isolates.

Technical Appendix Table 5. Capsular serotyping of *Capnocytophaga canimorsus* dog isolates by ELISA*

	Serovar J		Serovar K	
Isolate	Mean	SD	Mean	SD
H12	100	0	6.2	0.6
H24	10	2.2	100	0
Cc5	9.4	2.1	6.1	0.4
Cc6	9.6	2.3	6.4	0.5
Cc9	10	2.6	6.4	0.6
Cc12	9.7	2.3	7.5	1.0
Cc4	10	2.9	7.4	1.4
CcD3	6.9	0.7	5.5	0.4
CcD5	7.2	1.3	5.9	0.3
CcD6	7.6	1.8	6.4	0.8
CcD10	7.1	1.2	7.2	0.4
CcD13	6.4	1.2	5.7	0.5
CcD16	6.9	1.5	6.2	0.4
CcD18	8.4	2.1	6.9	0.7
CcD20	9.6	2.3	6.9	0.8
CcD25	6.6	1.1	6.0	0.4
CcD33	7.2	1.7	7.1	1.4
CcD34	8.7	2.0	6.4	1.9
CcD35	43.5	4.0	5.8	0.3
CcD37	6.2	1.3	5.6	0.2
CcD39	6.7	1.5	5.7	0.3
CcD40	7.4	1.8	7.0	1.3
CcD43	7.4	1.3	5.9	0.3
CcD44	6.3	1.2	5.4	0.2
CcD47	7.0	1.9	5.8	0.3
CcD51	7.6	2.0	6.4	1.0
CcD52	8.3	1.5	7.0	1.9
CcD53	7.6	1.9	6.3	0.4
CcD57	7.3	1.9	7.2	3.0
CcD58	9.9	4.1	6.8	0.8
CcD63	7.2	1.1	5.8	0.2
CcD68	8.0	2.8	5.8	0.4
CcD69	8.6	3.1	5.6	0.4
CcD71	8.8	2.0	5.7	0.4
CcD73	7.1	1.4	6.9	1.3
CcD76	6.8	1.3	5.4	0.2
CcD77	7.6	1.6	6.7	0.5
CcD80	7.3	1.4	6.2	0.6
CcD81	7.6	0.6	8.2	2.3
CcD84	7.6	1.0	7.1	0.5
CcD89	8.5	1.3	8.0	0.7
CcD96	7.6	1.0	12.4	8.3
CcD101	9.3	1.8	12.2	8.7
CcD104	7.6	0.8	10.8	7.3
CcD105	8.7	1.5	8.4	2.9
CcD106	9.5	1.8	11.2	2.9
CcD113	7.5	1.1	11.2	7.5
CcD115	7.5	1.5	11.8	8.2
CcD116	8.0	1.6	10.1	5.8
CcD117	8.1	1.8	12.8	8.6
CcD118	7.2	1.1	11.6	7.3
CcD119	7.6	1.5	11.5	7.4
CcD120	7.4	1.6	12.4	9.6
CcD122	8.8	1.6	11.5	8.8
CcD124	7.2	1.2	12.2	9.4
CcD126	7.3	1.3	11.3	8.2
CcD129	8.1	1.6	9.4	3.8
CcD130	8.4	1.6	14.7	10.2
CcD131	7.1	1.8	12.2	9.2

*Capsular serotyping was determined by ELISA on entire heat-killed bacteria. The following sera were used: anti-H12 adsorbed with human isolates Cc1 to Cc25 (J antiserum) and anti-H24 adsorbed with human isolates Cc1 to Cc25 (K antiserum). Isolates Cc5, Cc6, Cc9, Cc12, and Cc4, which were serovars A–E, respectively, were used as negative controls. The readout of the ELISA was absorbance but results are expressed here as percentage of reactivity calculated with respect to the absorbance value obtained for the capsular type strain. Values are the mean and SD of at least 3 independent experiments.

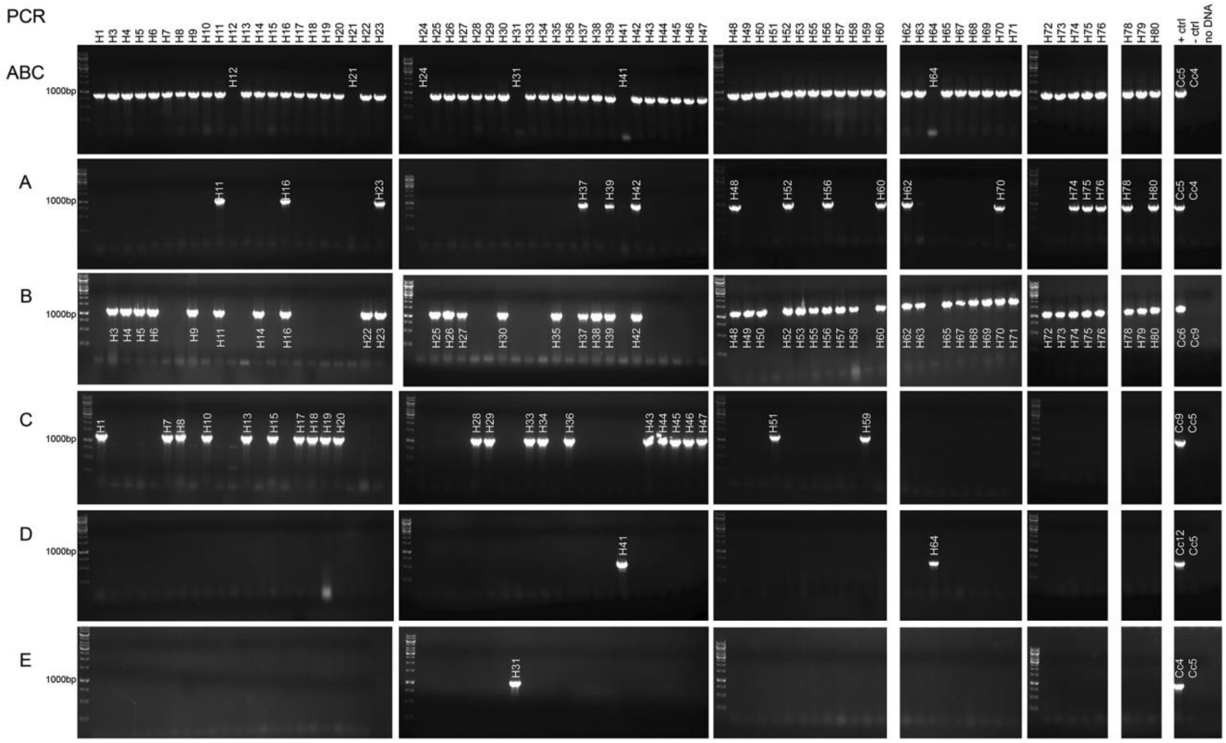

Technical Appendix Figure 1. Capsular typing of *Capnocytophaga canimorsus* isolates from patients, Helsinki, Finland, 2000–2017, by PCR. PCR detection of capsular serovars A–E with oligonucleotides given in Technical Appendix Table 2. The capsular type of each serovar described in reference (*10*) were used as positive controls: Cc5, Cc6 (*6*), Cc9 (*7*), Cc12 (American Type Culture Collection 35979) (*8*), and Cc4 (*4*) for A, B, C, D, and E serovars, respectively. For PCR ABC, Cc5 was used as a positive control. A strain of another capsular type and no DNA were used as negative controls. Three strains were identified by 16S rDNA sequencing to be a member of other dog-hosted *Capnocytophaga* species and were removed from our study and from this figure by cutting out the according lanes. Ctrl, control.

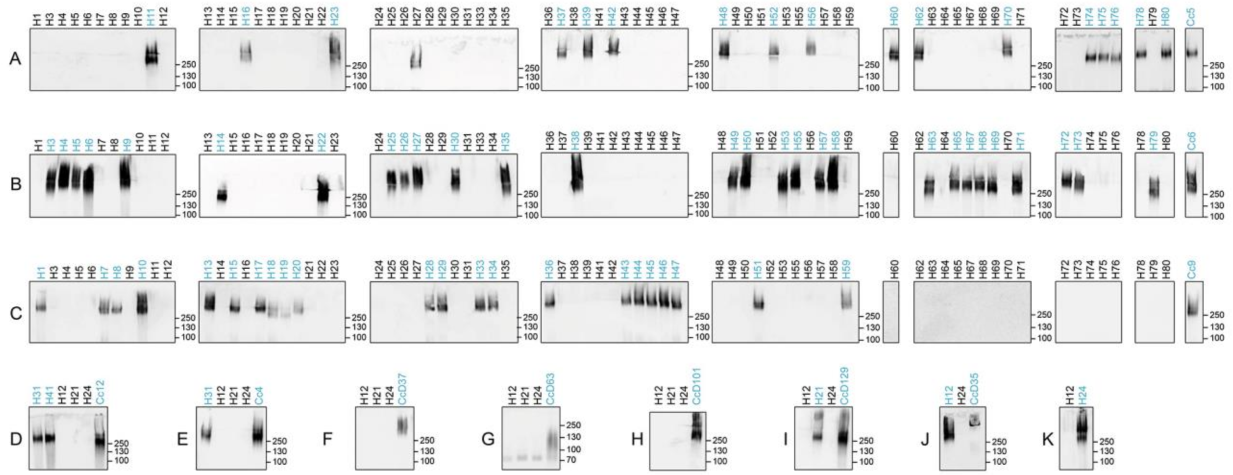

Technical Appendix Figure 2. Capsular serotyping of *Capnocytophaga canimorsus* isolates from patients, Helsinki, Finland, 2000–2017, by Western blot. Western blot analysis of proteinase-K treated lysates of *C. canimorsus* isolates by using A–K antisera. Cc5, Cc6, Cc9, Cc12, Cc4, CcD37 (*6*), CcD63 (*6*), CcD101 (*6*), and CcD129 (*6*) were used as positive controls for capsular serovars A–I, respectively. Numbers correspond to molecular mass (kDa). Three strains were identified by 16S rDNA sequencing to be members of other dog-hosted *Capnocytophaga* species and were removed from our study and from this figure by cutting out the according lanes.

References

1. Heltberg O, Busk HE, Bremmelgaard A, Kristiansen JE, Frederiksen W. The cultivation and rapid enzyme identification of DF-2. Eur J Clin Microbiol. 1984;3:241–3. PubMed http://dx.doi.org/10.1007/BF02014893

2. Hantson P, Gautier PE, Vekemans MC, Fievez P, Evrard P, Wauters G, et al. Fatal *Capnocytophaga canimorsus* septicemia in a previously healthy woman. Ann Emerg Med. 1991;20:93–4. PubMed http://dx.doi.org/10.1016/S0196-0644(05)81130-8

3. Vanhonsebrouck AY, Gordts B, Wauters G, Van Landuyt HW. Fatal septicemia with *Capnocytophaga canimorsus* in a compromised host. A case report with review of the literature. Acta Clin Belg. 1991;46:364–70. PubMed http://dx.doi.org/10.1080/17843286.1991.11718192

4. Rougemont M, Ratib O, Wintsch J, Schrenzel J, Hirschel B. *Capnocytophaga canimorsus* prosthetic aortitis in an HIV-positive woman. J Clin Microbiol. 2013;51:2769–71. PubMed http://dx.doi.org/10.1128/JCM.00321-13

5. Shin H, Mally M, Kuhn M, Paroz C, Cornelis GR. Escape from immune surveillance by *Capnocytophaga canimorsus*. J Infect Dis. 2007;195:375–86. PubMed http://dx.doi.org/10.1086/510243

6. Renzi F, Dol M, Raymackers A, Manfredi P, Cornelis GR. Only a subset of *C. canimorsus* strains is dangerous for humans. Emerg Microbes Infect. 2016;5:e29. PubMed http://dx.doi.org/10.1038/emi.2016.43

7. Vandamme P, Vancanneyt M, van Belkum A, Segers P, Quint WG, Kersters K, et al. Polyphasic analysis of strains of the genus *Capnocytophaga* and Centers for Disease Control group DF-3. Int J Syst Bacteriol. 1996;46:782–91. PubMed http://dx.doi.org/10.1099/00207713-46-3-782

8. Butler T, Weaver RE, Ramani TK, Uyeda CT, Bobo RA, Ryu JS, et al. Unidentified gram-negative rod infection. A new disease of man. Ann Intern Med. 1977;86:1–5. PubMed http://dx.doi.org/10.7326/0003-4819-86-1-1

9. Kleijnen-Grebien B, Boorsma S, Stals FS, van Schelven R. Fatal case of sepsis with *Capnocytophaga canimorsus* after a minor dog bite [in Dutch]. Ned Tijdschr Geneeskd. 2008;152:1882–5. PubMed

10. Hess E, Renzi F, Koudad D, Dol M, Cornelis GR. Identification of virulent *Capnocytophaga canimorsus* isolates by capsular typing. J Clin Microbiol. 2017;55:1902–14. PubMed http://dx.doi.org/10.1128/JCM.00249-17

11. Kotilainen P, Jalava J, Meurman O, Lehtonen OP, Rintala E, Seppälä OP, et al. Diagnosis of meningococcal meningitis by broad-range bacterial PCR with cerebrospinal fluid. J Clin Microbiol. 1998;36:2205–9. PubMed

12. Edwards KJ, Logan JM, Langham S, Swift C, Gharbia SE. Utility of real-time amplification of selected 16S rRNA gene sequences as a tool for detection and identification of microbial signatures directly from clinical samples. J Med Microbiol. 2012;61:645–52. PubMed http://dx.doi.org/10.1099/jmm.0.041764-0

13. Johnson JL. Similarity analysis of rRNAs. In: Gerhard P, Murray RGE, Wood NR, Krieg NR, editors. Methods for general and molecular bacteriology. Washington, DC (USA): American Society for Microbiology; 1994. p. 683–700.

14. Lane DJ. 16S/23S rRNA sequencing. In: Stackebrandt EG, Goodfellow M, editors. Nucleic acid techniques in bacterial systematics. New York (USA): John Wiley & Sons; 1991. p. 115–74.
